# Performance of 5 AI Models on United States Medical Licensing Examination Step 1 Questions: Comparative Observational Study

**DOI:** 10.2196/76928

**Published:** 2026-03-09

**Authors:** Dania El Natour, Mohamad Abou Alfa, Ahmad Chaaban, Reda Assi, Toufic Dally, Bahaa Bou Dargham

**Affiliations:** 1 Faculty of Medicine Beirut Arab University Beirut Lebanon; 2 Department of Anesthesiology Hammoud Hospital University Medical Center Saida Lebanon

**Keywords:** artificial intelligence, case-based reasoning, ChatGPT, Copilot, DeepSeek, Gemini, Grok, image-based questions, large language models, medical education, USMLE

## Abstract

**Background:**

Artificial intelligence (AI) models are increasingly being used in medical education. Although models like ChatGPT have previously demonstrated strong performance on United States Medical Licensing Examination (USMLE)–style questions, newer AI tools with enhanced capabilities are now available, necessitating comparative evaluations of their accuracy and reliability across different medical domains and question formats.

**Objective:**

This study aimed to evaluate and compare the performance of 5 publicly available AI models: Grok, ChatGPT-4, Copilot, Gemini, and DeepSeek, on the USMLE Step 1 free 120-question set, assessing their accuracy and consistency across question types and medical subjects.

**Methods:**

This cross-sectional observational study was conducted between February 10 and March 5, 2025. Each of the 119 USMLE-style questions (excluding 1 audio-based item) was presented to each AI model by using a standardized prompt cycle. Models answered each question 3 times to assess confidence and consistency. Questions were categorized as text-based or image-based and as case-based or information-based. Statistical analysis was performed using chi-square and Fisher exact tests, with Bonferroni adjustment for pairwise comparisons.

**Results:**

Grok achieved the highest score (109/119, 91.6%), followed by Copilot (101/119, 84.9%), Gemini (100/119, 84%), ChatGPT-4 (95/119, 79.8%), and DeepSeek (86/119, 72.3%). DeepSeek’s lower score was due to an inability to process visual media, resulting in 0% accuracy on image-based items. When limited to text-only questions (n=96), DeepSeek’s accuracy increased to 89.6% (86/96), matching Copilot. Grok showed the highest accuracy on image-based (21/23, 91.3%) and case-based questions (70/78, 89.7%), with statistically significant differences observed between Grok and DeepSeek on case-based items (*P*=.01). The models performed best in biostatistics and epidemiology (5.8/6, 96.7%) and worst in musculoskeletal, skin, and connective tissue (4.4/7, 62.9%). Grok maintained 100% consistency in responses, while Copilot demonstrated the most self-correction (112/119, 94.1% consistency), improving its accuracy to 89.9% (107/119) on the third attempt.

**Conclusions:**

AI models showed varying strengths across domains, with Grok demonstrating the highest accuracy and consistency in this dataset, particularly for image-based and reasoning-heavy questions. Although ChatGPT-4 remains widely used, newer models like Grok and Copilot also performed competitively. Continuous evaluation is essential as AI tools rapidly evolve.

## Introduction

Artificial intelligence (AI) technology enables machines to replicate a range of human abilities, such as learning, reasoning, and problem-solving [1]. AI has transformed various fields, including health care, where it has significantly impacted both clinical practice and education. Advances in large language models (LLMs), such as ChatGPT and others, have opened new possibilities in advancing medical education and practice [2]. A recent study examined ChatGPT-4’s diagnostic and triage accuracy and found performance comparable to physicians [3]. Similarly, Li et al [4] highlighted how LLMs are reshaping educator roles and redefining teaching and assessment strategies in medical education. It has accelerated diagnostic processes and clinical decision-making in several fields, such as radiology, pathology, endoscopy, and ultrasonography. AI models are showing promising capabilities in disease diagnosis and medical knowledge assessment, suggesting their growing role in both clinical and educational settings [5].

AI models, such as ChatGPT, have demonstrated significant benefits in answering medical questions across diverse disciplines, including medical biochemistry, gross anatomy, physiology, physical rehabilitation, surgery, neuroscience, and orthopedics, among many others [6-12]. Additionally, AI models have shown promising results in various national board examinations, including the United States Medical Licensing Examination (USMLE) and other exams in Thailand, China, and Japan, which are crucial for assessing medical knowledge and clinical decision-making [13-15]. Evaluations of LLMs such as ChatGPT-3.5, ChatGPT-4, and Bard have shown their ability to perform well in different medical exams, including the Multi-Specialty Recruitment Assessment and National Eligibility Entrance Test [16,17]. Likewise, consistent cross-exam accuracy patterns have been observed between ChatGPT and Bard across multiple international licensing examinations, including the USMLE, Professional and Linguistic Assessments Board Test, Hong Kong Medical Licensing Examination, and National Medical Licensing Examination [18]. Moreover, in the Turkish Medical Specialization Examination, ChatGPT-4 outperformed other models, underscoring the potential of AI in medical education and assessment [19].

One area of particular interest is the performance of AI models on USMLE-style questions (Step 1, Step 2, and Step 3) [20]. Evaluating AI models on these exams provides valuable insights into their potential as educational tools and highlights their limitations in addressing complex medical concepts. AI models have demonstrated notable accuracy across various medical domains, including basic and clinical sciences, and even shelf examinations. Among these, ChatGPT-4 has shown particularly high performance, consistently outperforming earlier models like ChatGPT-3.5 on USMLE-style questions [21]. For instance, ChatGPT-4 achieved a 71.33% accuracy on these questions, surpassing both ChatGPT-3.5 and human test-takers from AMBOSS [22]. It also excelled in soft skills assessments, answering 90% of questions related to ethical decision-making [23]. Other models, like Bing Chat, have also shown strengths in clinical problem-solving [13].

Earlier studies excluded image-based questions due to the limited visual processing capabilities of AI models. However, newer models like ChatGPT-4 have overcome this limitation and are now capable of handling larger word limits, solving more complex problems, and interpreting images [24]. As a result of these advancements, ChatGPT-4 continues to achieve the highest accuracy across various evaluations. However, models can exhibit variability in performance when asked the same question multiple times, suggesting that a single response may not always be sufficient for precise decision-making [9].

Despite the growing body of literature on the use of AI models in medical education, several gaps remain in understanding their effectiveness across different subjects and contexts. While AI models such as ChatGPT-4 and ChatGPT-3.5 have shown promise in answering questions related to medical knowledge and licensing exams, limited research has specifically focused on their performance within basic sciences such as pharmacology, biochemistry, and molecular biology. Another notable gap is the examination of AI models' capabilities in answering image-based medical questions. Although models like ChatGPT-4 have demonstrated potential in this area, no comprehensive evaluations have been conducted.

The National Board of Medical Examiners (NBME) Free 120 questions offer a comprehensive approach to medical education, covering topics such as basic sciences, body systems, ethics, and statistics, all of which are essential for licensing exams. Many previous studies have examined these topics in isolation, rather than as part of a unified assessment. Additionally, few studies have compared the performance of multiple AI models on the same set of questions.

Building upon these gaps, this study provides one of the first head-to-head evaluations of five contemporary AI models: Grok, ChatGPT-4, Copilot, Gemini, and DeepSeek, using the 2024 NBME Free 120-question set. Unlike previous studies, it simultaneously assesses performance across text-based and image-based items, as well as case-based vs information-based questions, and introduces the evaluation of response consistency across repeated prompts as a new indicator of model reliability. This multidimensional approach offers an updated benchmark for understanding how next-generation AI models perform in medical knowledge assessment.

The main goal of this study was to assess and compare the performance of these 5 AI models on the 2024 NBME Free 120-question set, which serves as a proxy for the USMLE Step 1 examination. Specifically, we aimed to measure their accuracy across diverse medical domains, spanning both basic science knowledge and clinical reasoning, while evaluating their ability to interpret image-based questions and maintain consistency across repeated responses.

## Methods

### Study Design and Setting

#### Overview

This study is a cross-sectional, observational, comparative analysis conducted in an online simulated environment between February 10 and March 5, 2025. We evaluated the performance of 5 openly accessible AI models: ChatGPT-4.0 (OpenAI), Copilot (Microsoft), Gemini 2.0 Flash (Google), Grok 2 (xAI), and DeepSeek (version 3; DeepSeek AI), on the 2024 NBME Free 120-question set, simulating the USMLE Step 1 exam. Note that only basic features of each AI model were used. This was achieved by creating only a standard account and using it as it would be used in everyday use, showcasing the availability and easy accessibility aspect of the study. To the best of our knowledge, at the time of data collection, use of publicly available examination-style questions for research purposes did not violate the publicly stated terms of service of the evaluated AI platforms.

#### Question Set

The question set used in this study was the USMLE Step 1 free sample test questions, provided in PDF format by the official USMLE website (January 2024 version) [24]. While the original set contained 120 multiple-choice questions, 1 cardiology question involving heart auscultation was excluded, as the audio component was not accessible in the PDF format and therefore could not be evaluated by the AI models. No other text-based question was dropped. As a result, a total of 119 questions were used for analysis.

All remaining questions were included, even those that did not require scientific medical knowledge, to ensure a holistic representation of the exam. The questions were categorized into 14 subject areas, similar to the organization used in commercial question-bank platforms. These subjects included musculoskeletal, skin, and connective tissue; respiratory; renal and urinary system; pharmacology; microbiology and immunology; neurology and psychiatry; gastroenterology; biochemistry and molecular biology; cardiovascular; biostatistics and epidemiology; ethics and communication skills; endocrinology; hematology and oncology; and reproduction.

In addition to subject categorization, the questions were also classified by question format: (1) Text-based vs visual media: visual media questions included imaging scans, histological sections, line graphs, organ cross-sections, blood smears, anatomical abnormalities, and pedigrees. Text-based questions contained no supplementary images. (2) Case-based vs information-based: case-based questions presented clinical scenarios requiring analysis, diagnosis, or decision-making. Information-based questions assessed factual recall, definitions, or straightforward concepts.

Each question was carefully reviewed for completeness and clarity before inclusion. Text-based items were formatted uniformly to remove extraneous spaces or symbols, while image-based questions were extracted directly from the original NBME PDF and presented identically across all models as PNG files for compatibility, without any preprocessing, cropping, or resolution modification. No manual editing of question content or answer options was performed to preserve authenticity.

#### Question Cycle and Assessment

Each of the 119 multiple-choice questions was presented individually to each AI model following a standardized prompt structure. Every USMLE-style question was preceded by the instruction: “This is a board-level exam for medical students. Read the question carefully and choose the correct answer.” This introductory phrase was provided on a separate line to simulate a testing environment and set a consistent tone.

Following the model’s initial response, it was prompted twice with the question: “Are you sure?” regardless of whether the answer was correct. This resulted in 3 responses per question, forming a structured interaction cycle defined as: Introductory phrase → USMLE question → Initial answer → Confidence reassessment (×2).

A simple prompting strategy was intentionally selected to simulate realistic interactions by medical students without prior experience in prompt engineering. This approach aimed to reflect how everyday users might use AI tools in practical study settings rather than under optimized experimental conditions. The phrase “Are you sure?” was used twice per item to prompt the model to self-reflect and verify its initial response, minimizing the likelihood of internal contradiction while maintaining a standardized and reproducible dialogue format across all models.

After each question cycle, a new chat log was created before proceeding to the next question to avoid any memory retention or carryover bias. This process was repeated for all 119 questions across each of the 5 AI models.

Regarding image-based questions, at the time of evaluation, all 5 models allowed image input through their publicly accessible interfaces; however, usage limits on the number of images per time period applied to free-tier access. To ensure consistency, image-based questions were submitted only after usage limits were reset. Biomedical images could be uploaded to all models; however, DeepSeek did not process visual input and therefore did not generate responses for image-based questions, reflecting an inherent lack of multimodal capability in the version tested.

Responses were recorded and organized into separate Excel sheets for each model. For every question, all 3 responses were logged and classified as either “correct” or “incorrect” based on the official answer key provided with the USMLE Free 120 sample. Accuracy was calculated as the percentage of correct responses at the initial attempt.

Consistency was evaluated by comparing the 3 consecutive answers given by each model to assess how often responses changed. This provided insight into each model’s internal confidence and its tendency to revise answers.

Finally, the performance of the AI models was analyzed in terms of accuracy, consistency, and performance across subjects and question types (text-based vs visual; case-based vs information-based). Examples of the full prompt cycles are available in Multimedia Appendix 1.

### AI Model Selection and Inclusion Criteria

This study did not involve human participants. Instead, the analysis was based on AI-generated responses from 5 LLMs: ChatGPT-4.0 (OpenAI), Copilot (Microsoft), Gemini 2.0 Flash (Google), Grok 2 (xAI), and DeepSeek (version 3; DeepSeek AI). These models were selected based on their public accessibility, widespread use, and availability without requiring premium subscriptions or special configurations. Only the free, publicly accessible versions of each AI model were used during the data collection period to ensure equity in comparison.

Each AI model was independently tested using the same set of standardized prompts. All settings, including interface refresh between questions, were applied consistently across models to ensure methodological uniformity. No additional plug-ins, browser extensions, or special application programming interfaces were used.

### Bias Minimization

Efforts were made to minimize potential sources of bias throughout the study design and execution. To reduce information bias, all AI models were presented with the exact same prompts, question order, and formatting using a standardized prompt cycle. To avoid automation bias and assess confidence, each model was asked, “Are you sure?” twice after its initial response.

Selection bias was mitigated by including a diverse set of 5 widely-used, publicly accessible AI models, without restricting the sample to models requiring subscription or professional use. Additionally, a new chat log was created after each question cycle to eliminate any influence from retained memory or chat history.

All answers were manually reviewed and labeled as correct or incorrect using the official NBME answer key to avoid measurement bias. No plug-ins, enhancements, or adjustments were made to any model, ensuring that each was evaluated under equivalent and unbiased conditions.

### Study Size

The study size was determined by the number of questions available in the publicly released USMLE Step 1 free 120-question sample set (January 2024 version), provided by the NBME [25]. One question involving auscultation audio could not be evaluated due to the lack of accessible sound files in the PDF format and was therefore excluded. The final study sample comprised 119 multiple-choice questions, all of which were included in the analysis. A formal sample size calculation was not conducted, as the study was designed to evaluate AI model performance across the full breadth of a standardized and widely recognized medical exam sample.

### Data Analysis

#### Overview

All variables of interest, mainly accuracy, consistency, question type, and subject category, were assessed using a standardized process. The data source for question content was the publicly available NBME Free 120 (January 2024 version), which provided uniform test material. Each AI model received the same questions, in the same order, using an identical prompting structure. All responses were manually recorded and categorized by a single reviewer to minimize variability. No AI model received additional information, tools, or adjustments, ensuring full comparability across groups in terms of data exposure and measurement conditions.

#### Statistical Methods

Descriptive statistics were used to summarize the overall performance of each AI model in terms of accuracy and consistency. Accuracy was calculated as the percentage of correctly answered questions at the first attempt, while consistency was defined as the percentage of unchanged answers across 3 consecutive responses.

Differences in performance among AI models were analyzed using the chi-square test for categorical comparisons. Where expected cell counts were low, the Fisher exact test was used instead. The Bonferroni correction was applied to adjust for multiple pairwise comparisons between models across different question categories and subject domains. Only post hoc pairwise *P* values were Bonferroni-adjusted, whereas overall omnibus chi-square *P* values were presented as unadjusted.

No confounder adjustment was necessary, as the study did not include human participants or demographic variables. All AI models were exposed to the same set of questions under identical conditions, minimizing the potential for confounding.

All statistical analyses were conducted using SPSS Statistics software (version 29; IBM Corp). The analysis plan was determined a priori based on the study objectives. No sampling strategy was applied, as all available questions were included and equally presented to each AI model.

#### Additional Analyses

Subgroup analyses were conducted to evaluate the performance of AI models across different question formats (text-based vs image-based), question types (case-based vs information-based), and medical subject categories (14 domains). These subgroup comparisons were prespecified based on the structure of the NBME question set and the study objectives.

No formal interaction terms or sensitivity analyses were conducted, as the study’s primary aim was descriptive performance comparison rather than modeling effect modification. Given the highly standardized design, where all AI models were exposed to the same fixed set of questions under identical prompting conditions, and the absence of confounders or complex modeling assumptions, additional analyses were not deemed necessary.

### Ethical Considerations

This study did not involve human participants, patient data, or identifiable personal information. The dataset consisted exclusively of publicly available USMLE Step 1 examination-style questions obtained from the official NBME website. No human participants were recruited, and no personal or clinical data were accessed or analyzed. According to institutional guidelines, formal institutional review board approval was not required for this type of study. Informed consent was not required, as no individuals were enrolled or involved in the research process. All analyses were conducted in accordance with principles of research integrity and transparency.

## Results

### Overview

**Figure 1:**
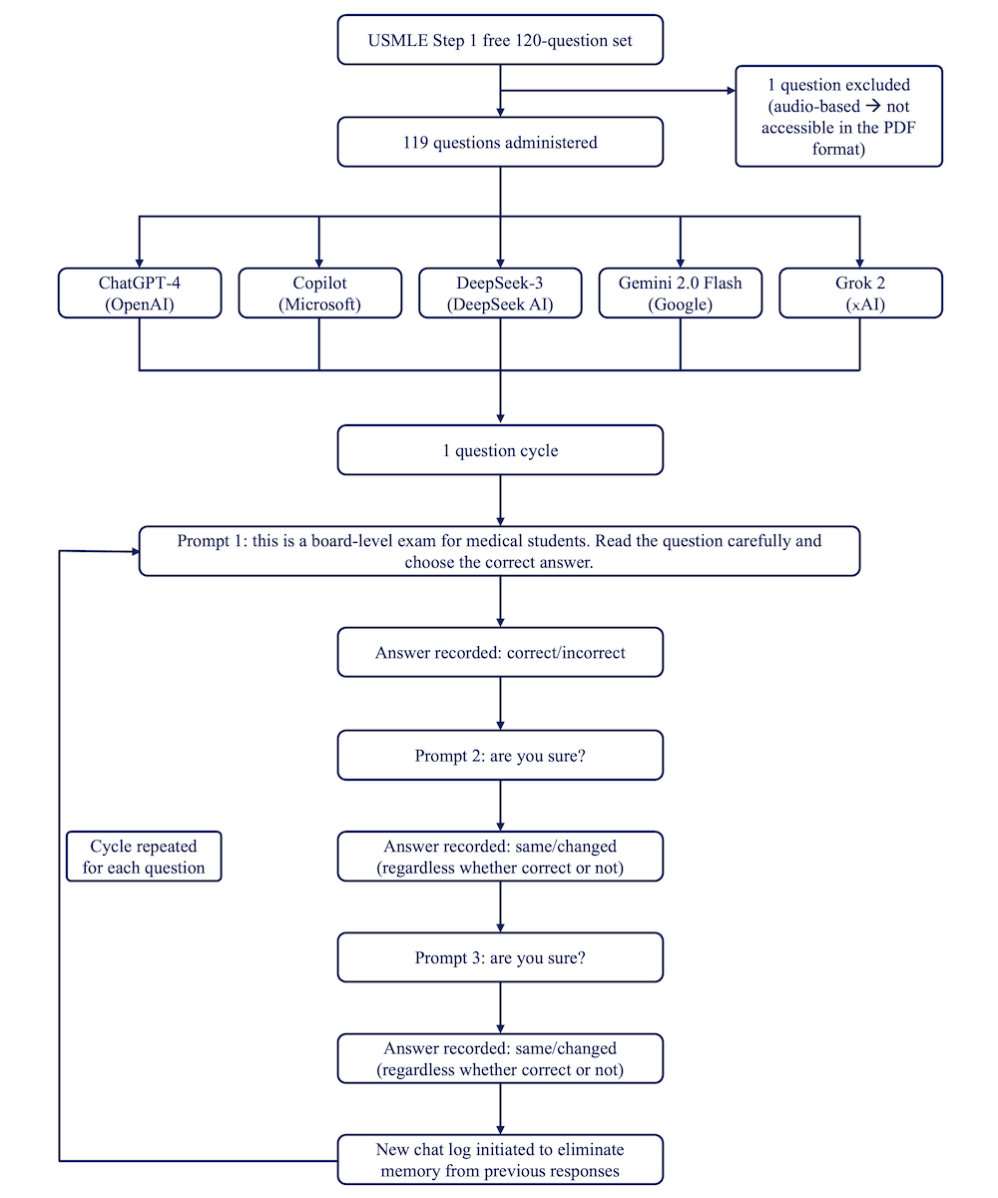
Flow diagram of artificial intelligence model testing procedure. USMLE: United States Medical Licensing Examination.

The inclusion of AI models and the question dataset used have been detailed in the Methods section. The flow of AI model testing, including the number of questions administered and prompt cycle structure, is summarized in Figure 1.

### Descriptive Overview of Question Set

The question set used in this study, administered to each AI model, consisted of the publicly available USMLE Step 1 free sample test questions (January 2024 version) in PDF format [24]. A total of 119 multiple-choice questions were included in the analysis after excluding 1 auscultation-based item due to a lack of audio accessibility.

Of these questions, 96 (80.7%) were text-only, while 23 (19.3%) included visual media, such as clinical images, pathology slides, histology sections, or diagnostic graphs. Questions were also categorized by format, with 78 (65.5%) classified as case-based and 41 (34.5%) as information-based.

The dataset spanned a broad range of 14 medical subject areas, consistent with the structure of commercial USMLE-style question banks. A detailed breakdown of question distribution by type, format, and subject is provided in Table 1.

**Table 1:** Distribution of United States Medical Licensing Examination Step 1 questions by type, format, and subject area (N=119).

Group and subgroup		Questions, n (%)	
**Type of question**			
	Text only	96 (80.7)	
	With visual media	23 (19.3)	
**Question format**			
	Information-based	41 (34.5)	
	Case-based	78 (65.5)	
**Subjects**			
	Biochemistry and molecular biology	7 (5.9)	
	Biostatistics and epidemiology	6 (5)	
	Cardiovascular	8 (6.7)	
	Endocrinology	7 (5.9)	
	Ethics and communication skills	9 (7.6)	
	Gastrointestinal	14 (11.8)	
	Hematology and oncology	9 (7.6)	
	Microbiology and immunology	8 (6.7)	
	Musculoskeletal, skin, and connective tissue	7 (5.9)	
	Neurology, special senses, and psychiatry	10 (8.4)	
	Pharmacology	6 (5)	
	Reproduction	10 (8.4)	
	Respiratory	6 (5)	
	Uro-renal	12 (10.1)	

### Results by Model Accuracy, Consistency, and Question Type

#### Overall Performance

Grok achieved the highest overall accuracy, correctly answering 91.6% (109/119) of the questions, followed by Copilot (101/119, 84.9%), Gemini (100/119, 84%), ChatGPT-4 (95/119, 79.8%), and DeepSeek, which had the lowest performance at 72.3% (86/119; Table 2). This distribution suggests a clear performance gradient among the models. A chi-square analysis revealed a statistically significant difference between the groups (*P*=.002), with pairwise comparisons identifying a significant difference specifically between Grok and DeepSeek, whose performance gap was substantial.

**Table 2:** Performance accuracy and answer consistency across artificial intelligence tools over 3 attempts on United States Medical Licensing Examination Step 1 questions (N=119).

		ChatGPT, n (%; 95% CI)	Copilot, n (%; 95% CI)	DeepSeek, n (%; 95% CI)	Gemini, n (%; 95% CI)	Grok, n (%; 95% CI)	*P* value	Effect size^a^	Post hoc^b^
**Attempt^c^**									
	First	95 (79.8; 72.8-86.7)	101 (84.9; 78.8-90.9)	86 (72.3; 64.3-80.2)	100 (84; 77.7-90.3)	109 (91.6; 86.4-96.8)	.002	0.084	Grok > DeepSeek
	Second	95 (79.8; 72.8-86.7)	100 (84; 77.7-90.3)	85 (71.4; 63.4-79.4)	100 (84; 77.7-90.3)	109 (91.6; 86.4-96.8)	.001	0.086	Grok > DeepSeek
	Third	96 (80.7; 73.9-87.5)	107 (89.9; 84.5-95.4)	86 (72.3; 64.3-80.2)	100 (84; 77.7-90.3)	109 (91.6; 86.4-96.8)	<.001	0.094	(Grok=Copilot) > DeepSeek
**Consistency**									
	No change in answer	118 (99.2; 97.7-100)	112 (94.1; 89.2-99)	115 (96.6; 92.9-100)	117 (98.3; 95.9-100)	119 (100; 100-100)	.02	0.069	No significant pairwise difference (after Bonferroni adjustment)

^a^Effect size is reported using Cramér V, which measures the strength of association between categorical variables (0.10=small, 0.30=medium, and 0.50=large).

^b^*P* values for post hoc pairwise comparisons were Bonferroni-adjusted to control for multiple testing. Omnibus chi-square *P* values are unadjusted. *P*<.05 is statistically significant.

^c^The first, second, and third attempts correspond to the initial answer, the response after the first confirmation prompt “Are you sure?,” and the response after the second confirmation prompt “Are you sure?,” respectively.

It is important to note that DeepSeek was unable to process questions that included visual media and thus defaulted to null responses for all image-based items. When these 23 questions were excluded from the dataset (leaving 96 text-only questions), DeepSeek’s accuracy increased to 89.6% (86/96), equaling that of Copilot. In this subset, Grok and Gemini both achieved the highest accuracy at 91.7% (88/96). However, the difference among models was not statistically significant in this analysis (*P*=.13; Table 3).

**Table 3:** Accuracy of artificial intelligence tools in answering United States Medical Licensing Examination Step 1 questions by type, format, and subject area.

		ChatGPT, n (%; 95% CI)	Copilot, n (%; 95% CI)	DeepSeek, n (%; 95% CI)	Gemini, n (%; 95% CI)	Grok, n (%; 95% CI)	*P* value^a^	Post hoc
**Question type**								
	Text only (n=96)	78 (81.3; 72.3-87.8)	86 (89.6; 81.9-94.2)	86 (89.6; 81.9-94.2)	88 (91.7; 84.4-95.7)	88 (91.7; 84.4-95.7)	.13	N/A^b^
	With visual media (n=23)	17 (73.9; 53.5-87.5)	15 (65.2; 44.9-81.2)	0 (0; 0-14.3)	12 (52.2; 33-70.8)	21 (91.3; 73.2-97.6)	<.001	All > DeepSeek Grok > Gemini
**Question format**								
	Information-based (n=41)	33 (80.5; 66-89.8)	35 (85.4; 71.6-93.1)	33 (80.5; 66-89.8)	37 (90.2; 77.5-96.1)	39 (95.1; 83.9-98.7)	.23	N/A
	Case-based (n=78)	62 (79.5; 69.2-87)	66 (84.6; 75-91)	53 (67.9; 57-77.3)	63 (80.8; 70.7-88)	70 (89.7; 81-94.7)	.01	Grok > DeepSeek
**Subject**								
	Biochemistry and molecular biology (n=7)	6 (85.7; 48.7-97.4)	7 (100; 64.6-100)	5 (71.4; 35.9-91.8)	6 (85.7; 48.7-97.4)	6 (85.7; 48.7-97.4)	.95	N/A
	Biostatistics and epidemiology (n=6)	6 (100; 61-100)	5 (83.3; 43.6-97)	6 (100; 61-100)	6 (100; 61-100)	6 (100; 61-100)	>.99	N/A
	Cardiovascular (n=8)	7 (87.5; 52.9-97.8)	7 (87.5; 52.9-97.8)	3 (27.5; 13.7-69.4)	6 (75; 40.9-92.9)	8 (100; 67.6-100)	.04	No significant pairwise difference (after Bonferroni adjustment)
	Endocrinology (n=7)	4 (57.1; 25-84.2)	6 (85.7; 48.7-97.4)	5 (71.4; 35.9-91.8)	7 (100; 64.6-100)	5 (71.4; 35.9-91.8)	.55	N/A
	Ethics and communication skills (n=9)	8 (88.9; 56.5-98)	7 (77.8; 45.3-93.7)	9 (100; 70.1-100)	8 (88.9; 56.5-98)	8 (88.9; 56.5-98)	.95	N/A
	Gastrointestinal (n=14)	12 (85.7; 60.1-96)	13 (92.9; 68.5-98.7)	9 (64.3; 38.8-83.7)	11 (78.6; 52.4-92.4)	12 (85.7; 60.1-96)	.47	N/A
	Hematology and oncology (n=9)	8 (88.9; 56.5-98)	7 (77.8; 45.3-93.7)	8 (88.9; 56.5-98)	8 (88.9; 56.5-98)	8 (88.9; 56.5-98)	>.99	N/A
	Microbiology and immunology (n=8)	7 (87.5; 52.9-97.8)	7 (87.5; 52.9-97.8)	6 (75; 40.9-92.9)	6 (75; 40.9-92.9)	8 (100; 67.6-100)	.85	N/A
	Musculoskeletal, skin, and connective tissue (n=7)	6 (85.7; 48.7-97.4)	5 (71.4; 35.9-91.8)	2 (28.6; 8.2-64.1)	3 (42.9; 15.8-75)	6 (85.7; 48.7-97.4)	.12	N/A
	Neurology, special senses, and psychiatry (n=10)	6 (60; 31.3-83.2)	8 (80; 49-94.3)	7 (70; 39.7-89.2)	10 (100; 72.2-100)	9 (90; 59.6-98.2)	.25	N/A
	Pharmacology (n=6)	3 (50; 18.8-81.2)	5 (83.3; 43.6-97)	5 (83.3; 43.6-97)	5 (83.3; 43.6-97)	5 (83.3; 43.6-97)	.76	N/A
	Reproduction (n=10)	7 (70; 39.7-89.2)	9 (90; 59.6-98.2)	7 (70; 39.7-89.2)	9 (90; 59.6-98.2)	10 (100; 72.2-100)	.30	N/A
	Respiratory (n=6)	5 (83.3; 43.6-97)	5 (83.3; 43.6-97)	5 (83.3; 43.6-97)	5 (83.3; 43.6-97)	6 (100; 61-100)	>.99	N/A
	Uro-renal (n=12)	10 (83.3; 55.2-95.3)	10 (83.3; 55.2-95.3)	9 (75; 46.8-91.1)	10 (83.3; 55.2-95.3)	12 (100; 75.8-100)	.59	N/A

^a^*P* values are based on chi-square or Fisher exact test, with Bonferroni adjustment applied in post hoc comparisons. Omnibus chi-square *P* values are unadjusted. *P*<.05 is statistically significant.

^b^N/A: not applicable.

#### Consistency Across Attempts

Each AI model was asked “Are you sure?” twice per question, regardless of the accuracy of its initial answer. Grok demonstrated perfect consistency (100%), with no changes in responses across all attempts. In contrast, Copilot revised its answers most frequently, yielding the lowest consistency rate at 94.1%.

While most models maintained similar accuracy across the 3 attempts, Copilot showed notable self-correction, improving from 84.9% to 89.9% on the third attempt. Despite this improvement, the overall ranking of models remained unchanged, with Grok and Copilot maintaining significantly higher accuracy than DeepSeek. Detailed results are provided in Table 2.

To better understand the direction of these revisions, the transitions between first and third attempts were analyzed (Table 4). Across models, most changes were beneficial, moving from incorrect to correct responses, while detrimental reversions were rare. Copilot showed the highest number of beneficial revisions (n=6), followed by DeepSeek (n=2). Grok maintained complete stability, with no changes across attempts.

**Table 4:** Accuracy across attempts and direction of answer changes between first and third attempts for each artificial intelligence model. Accuracy values represent the percentage of correct responses out of 119 questions per model. Transitions reflect changes between the first and third attempts. “Incorrect to correct” indicates beneficial revisions, while “correct to incorrect” denotes detrimental changes.

Model	Accuracy (first attempt), n (%)	Accuracy (third attempt), n (%)	Incorrect to correct, n	Correct to incorrect, n
ChatGPT	95 (79.8)	96 (80.7)	1	0
Copilot	101 (84.9)	107 (89.9)	6	0
DeepSeek	86 (72.3)	86 (72.3)	2	2
Gemini	100 (84)	100 (84)	1	1
Grok	109 (91.6)	109 (91.6)	0	0

#### Performance on Text vs Image-Based Questions

When evaluating only image-based questions (n=23), performance decreased for all models. Grok achieved the highest accuracy (21/23, 91.3%), followed by ChatGPT (17/23, 73.9%), Copilot (15/23, 65.2%), and Gemini (12/23, 52.2%). DeepSeek was unable to answer image-based questions, resulting in 0% (0/23) accuracy. Differences were statistically significant (*P*<.001), particularly between Grok and Gemini, and between DeepSeek and all other models, highlighting Grok’s superior image interpretation ability. No substantial performance changes occurred across the second and third attempts for image-based questions.

In contrast, for the 96 text-based questions, performance differences were not statistically significant (*P*>.05). Grok and Gemini achieved the highest scores (88/96, 91.7%), while ChatGPT had the lowest (78/96, 81.3%). Full results are summarized in Table 3.

#### Performance by Question Type: Case-Based vs Information-Based

For case-based questions, there was a statistically significant difference in performance between the AI models (*P*=.01), with Grok outperforming DeepSeek, as confirmed by pairwise comparison with Bonferroni adjustment. Grok achieved an accuracy of 89.7% (70/78), while DeepSeek scored 67.9% (53/78).

For information-based questions, Grok again led with 95.1% (39/41), followed by Gemini (37/41, 90.2%), Copilot (35/41, 85.4%), and both ChatGPT and DeepSeek (33/41, 80.5%). However, these differences were not statistically significant (*P*=.23).

Detailed breakdowns by question type are included in Table 3.

#### Performance by Medical Subject Area

Across the 14 medical domains, AI models showed no statistically significant differences in response behavior (*P*>.05). Although Grok achieved 100% accuracy in 6 domains, and DeepSeek performed worst in cardiovascular (3/8, 27.5%), pairwise comparisons did not remain significant after applying the Bonferroni correction.

When analyzing subject-wise accuracy across all models, the best-performing domains were: biostatistics and epidemiology (5.8/6, 96.7%), ethics and communication skills (8/9, 88.9%), and hematology-oncology (7.8/9, 86.7%). On the other hand, the lowest-performing domains were: pharmacology (4.6/6, 76.6%), cardiovascular (6.2/8, 75.5%), and musculoskeletal, skin, and connective tissue (4.4/7, 62.9%)

**Figure 2:**
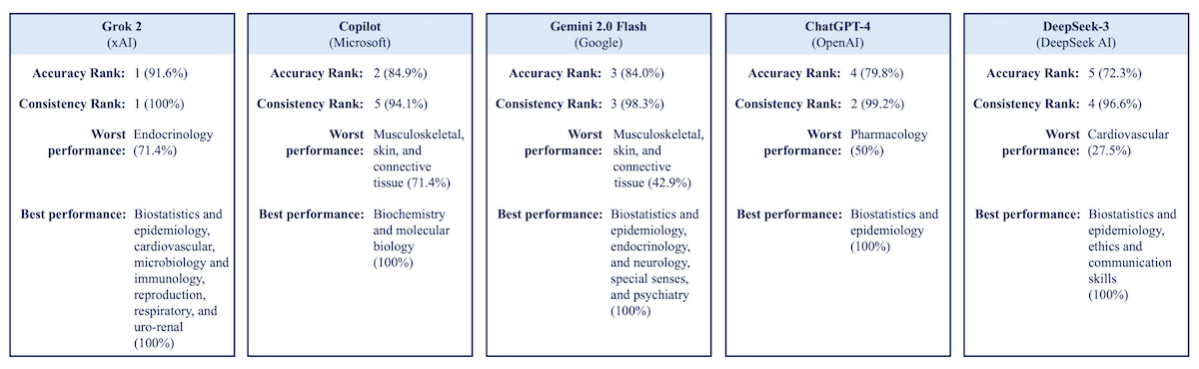
Individual artificial intelligence model profiles: accuracy, consistency, and subject-based strengths.

A summary of each AI model’s performance is presented in Figure 2, highlighting their accuracy and consistency rankings, best- and worst-performing subject areas, and overall strengths and limitations.

## Discussion

### Principal Results

#### Overview

This study provides one of the first direct comparisons among 5 contemporary AI models, Grok, ChatGPT-4, Copilot, Gemini, and DeepSeek, on a standardized USMLE-style dataset. Unlike earlier studies that primarily focused on ChatGPT or text-based questions, our analysis includes image- and case-based items and introduces response consistency as a new metric of reliability. These elements together offer a multidimensional view of model performance, expanding current understanding of how next-generation AI tools behave under realistic exam conditions.

In terms of performance, Grok achieved the highest overall accuracy (109/119, 91.6%) and demonstrated the strongest performance across image- and case-based questions within this evaluation framework. Copilot and Gemini followed closely, while ChatGPT-4, despite its popularity, ranked fourth. DeepSeek placed last overall, primarily due to the absence of image interpretation capability in the publicly available version at the time of testing (the image could be uploaded, but the model could not interpret it when combined with text or when presented alone, as the version tested did not support multimodal input). This limitation reflects the functionality at the time of evaluation and may not apply to future model updates. However, when excluding image-based content, DeepSeek performed comparably to Copilot. Across all models, the highest performance was noted in biostatistics and epidemiology, ethics and communication skills, and hematology-oncology, while the lowest scores occurred in pharmacology, cardiology, and musculoskeletal content.

ChatGPT is the AI model with the most internet traffic, receiving over 4.7 billion monthly visits [26]. Given its widespread use, it is crucial to evaluate its ability to answer the types of questions that medical students may encounter, as it plays a significant role in the evolution of medical education [27]. The finding that ChatGPT ranked below Grok, Copilot, and Gemini contrasts with much of the prior literature, which typically showed ChatGPT outperforming other models [18,21,24,28]. This shift in performance trends, with newer models like Grok demonstrating increasing accuracy, highlights the importance of continuously evaluating and adapting AI tools in medical education.

Although the models performed differently, there were no drastic changes in their answers when assessing their confidence, suggesting a degree of stability in their responses. Menekşeoğlu and İş [9] observed a change rate of 32.2% in ChatGPT-4, which contrasts with our findings, where ChatGPT-4 remained consistent in most of its answers. They also noted a 1% change in Gemini (formerly Bard), which aligns with our study, where Gemini demonstrated a high consistency rate [9]. Copilot showed the most significant changes in its answers when assessing its confidence (112/119, 94.1%), along with an improvement in accuracy, highlighting the importance of self-revision in some AI models.

The high consistency observed in our study, particularly in newer models, may indicate continuous updates and algorithmic improvements, suggesting these tools are becoming more stable and potentially more accurate over time. This trend underscores the importance of ongoing benchmarking and reassessment to track performance shifts and ensure that AI tools remain reliable and relevant for educational use.

The performance discrepancy between image-based and text-based questions was particularly noteworthy, underscoring the importance of visual reasoning in medical assessments. Notably, our finding for ChatGPT-4's performance on image-based questions (17/23, 73.9%) is somewhat higher than that reported in a previous study, where ChatGPT-4 achieved 63.2% accuracy [29]. While we did not perform a statistical comparison, the similarity in these results suggests a consistent trend in the model's moderate performance with visual inputs, and underscores the comparative advantage of newer models like Grok in this area.

All models performed better on information-based questions than on case-based ones, although the overall difference was not statistically significant. However, a significant difference was observed among models specifically for case-based questions (*P*=.01). This suggests that while AI models are generally proficient in retrieving factual information, they face greater challenges when required to analyze and reason through complex clinical scenarios.

Our findings align with those of Tosun et al [30], who also identified a noticeable drop in AI performance on case-based versus information-based questions within a dental education context [30]. Their study, like ours, revealed a consistent performance gap across all models, reinforcing the notion that tasks involving contextual understanding and clinical judgment remain particularly difficult for current AI systems. These findings underscore the ongoing need for further development and refinement of AI models, especially in managing complex, multimodal tasks encountered in real-world medical education and practice.

Beyond question format, we further examined performance across different medical subjects to explore topic-specific strengths and weaknesses of the AI models. These models demonstrated varying performance across medical subjects, reflecting the nature and complexity of the content within each domain. The highest scores were observed in biostatistics and epidemiology (5.8/6, 96.7%), ethics and communication skills (8/9, 88.9%), and hematology-oncology (7.8/9, 86.7%). These subjects tend to be more rule-based and structured, aligning well with the strengths of LLMs. Biostatistics questions often involve applying well-defined formulas and concepts, while ethics and communication scenarios are typically guided by standardized principles such as autonomy, consent, and confidentiality, commonly encountered in AI training data. Similarly, hematology-oncology involves recognizable patterns and standard management pathways, making it more accessible for AI models to navigate. These high scores suggest that structured, rule-based domains may currently be where AI performs most reliably. However, comparisons with existing literature reveal nuances in interpretation.

Our findings on ethics and communication skills contrast with the results of Khan et al [23], who reported that medical students generally outperform AI models in ethics questions [23]. Similarly, Chen et al [18] observed lower accuracy in AI responses within ethical contexts [18], emphasizing that while AI can apply general rules, it still lacks the empathy and nuanced understanding required for complex ethical scenarios. Likewise, our strong performance in hematology-oncology differs from the reports of Chen et al [18] of sub-70% accuracy in hematolymphoid questions, suggesting that either newer models have improved or differences in question style and format may explain the variation.

Conversely, lower performance was observed in pharmacology (4.6/6, 76.6%), cardiology (6.2/8, 77.5%), and musculoskeletal, skin, and connective tissue (4.4/7, 62.9%). These domains often require high precision, contextual interpretation, and image-based analysis. For instance, pharmacology questions demand detailed recall of mechanisms, dosages, and adverse effects, many of which involve similar-sounding drug names. Cardiology often includes stepwise clinical decision-making and visual inputs like electrocardiograms or echocardiograms, which can challenge models not designed for multimodal processing. The musculoskeletal and connective tissue category heavily depends on visual diagnostics (eg, skin lesions and radiographs) and clinical nuance, which likely contributed to the notably lower scores. Interestingly, our findings in cardiology align with the study by Penny et al [22], which reported a 56% accuracy rate in cardiovascular system questions. However, Penny et al [22] also noted an 85% accuracy in musculoskeletal questions, which contrasts with our much lower result of 62.9% (4.4/7), suggesting potential variability in question complexity or in the capabilities of different AI models.

These variations in accuracy highlight the need for further exploration into the consistency of AI models' performance across different medical topics. This may reflect either the complexity of the questions or inconsistencies in the models themselves, rather than inherent challenges with specific subject areas. Our results underscore the importance of conducting multiple trials when using AI models in multiple-choice question-based research. However, it is worth noting that subject-level comparisons were based on a limited number of questions per topic (6-14 questions), resulting in limited statistical power. Accordingly, these subject-specific findings should be interpreted as descriptive and exploratory rather than inferential, particularly as most comparisons did not remain statistically significant after Bonferroni correction.

Placed within the context of prior LLM-USMLE benchmarking studies, which have largely focused on single models and text-only evaluations, this work extends the literature by proposing a standardized, multimodal benchmarking framework and by evaluating response consistency as an indicator of AI self-correction behavior, with direct relevance to medical education implementation and informatics research.

Among the 5 AI models evaluated, Grok 2.0, developed by xAI, demonstrated the highest overall performance in this dataset, excelling in both text- and image-based questions. It consistently scored above 80% in nearly all subjects, except endocrinology (5/7, 71.4%), and was the only model to accurately interpret all visual inputs. While previous research showed that ChatGPT outperformed Grok-1 [31,32], Shiraishi et al [31] acknowledged that newer iterations like Grok 2.0 may surpass ChatGPT and Claude models. Given its accuracy and consistency, within the context of this evaluation, Grok may be suitable for certain educational applications. Microsoft Copilot also performed well, particularly in its ability to revise and improve its answers over multiple attempts. However, its inconsistent handling of visual media (sometimes refusing to analyze clinical images) contributed to a lower image-based score (15/23, 65.2%), suggesting a possible selection bias. Despite this, Copilot’s text-only accuracy reached 89.6% (86/96), tying with DeepSeek. These findings contrast with previous literature that typically reported ChatGPT outperforming Copilot in broader medical assessments [33,34].

Gemini (formerly Bard), developed by Google DeepMind, performed consistently across question types but showed limited capacity to interpret visual media, likely due to stricter internal filters. While Gemini achieved a perfect consistency rate, it scored only 52.2% (12/23) on image-based questions. Other studies have similarly found Copilot outperforming Gemini [33], although Gemini has matched ChatGPT’s accuracy in specific fields like ophthalmology [35]. Surprisingly, ChatGPT-4, despite being widely used and highly ranked in earlier studies [18,19,21,24,28], ranked second to last in our analysis. Still, it maintained a strong knowledge base and 99.2% (118/119) response consistency. Finally, DeepSeek, released in January 2025 by Hangzhou DeepSeek AI, exhibited the lowest overall accuracy score in this dataset, a finding driven by the absence of image interpretation capability rather than inferior reasoning performance. However, it excelled in the text-only category with an accuracy of 89.6% (86/96), comparable to Copilot. External evaluations on HuggingFace have shown DeepSeek-R1 outperforming ChatGPT-4 and OpenAI o1 in several benchmark tasks [36]. Its “Thinking Critically” feature may explain its high performance in factual recall. Addressing its image interpretation limitations could make DeepSeek a strong contender in medical education applications.

#### Implications for Medical Education and Practice

Recent advances in AI, particularly with LLMs such as ChatGPT-4, are transforming medical education and clinical practice by offering personalized learning tools, enhancing diagnostic accuracy, and refining standardized assessments [37]. These models are increasingly used to answer USMLE-style questions across diverse disciplines, functioning as virtual tutors that adapt to learners’ needs and simulate complex clinical scenarios [38]. The integration of AI tools into medical curricula has been met with enthusiasm from both medical students and educators, who recognize their potential to enhance accessibility, engagement, and individualized feedback [39-41]. In addition, LLMs such as ChatGPT-4 have demonstrated the ability to generate educational materials, including multiple-choice questions and “illness scripts,” that meet acceptable academic standards following expert review [42,43].

However, practical implementation requires careful consideration of potential drawbacks. Overreliance on AI tools may diminish cognitive engagement, problem-solving, and long-term retention among learners [44,45]. Excessive dependence may also encourage passive learning and erode the development of critical thinking and clinical reasoning skills essential for medical professionals [46,47]. Furthermore, AI models can occasionally produce confidently incorrect or fabricated (“hallucinated”) responses, underscoring the need for faculty oversight and structured guidance to ensure accuracy and responsible use. Therefore, successful integration into medical education should prioritize supervised use, explicit training on AI limitations, and continuous evaluation of its educational impact to ensure these tools complement, rather than replace, human judgment and expertise [48].

#### Relevance to Medical Informatics

Beyond the educational context, the findings of this study contribute to the evolving field of medical informatics by illustrating how LLMs can serve as dynamic tools within digital health and educational informatics systems. The comparative analysis highlights the variability in accuracy among current AI systems, emphasizing the importance of continuous benchmarking to ensure data integrity and reliability in informatics-driven applications. As medical informatics increasingly integrates AI for knowledge retrieval, automated assessment, and decision support, understanding model behavior across domains becomes essential for designing safe, adaptive, and transparent systems. The observed performance differences underscore the need for informatics standards that balance innovation with quality assurance, ensuring that AI-enhanced platforms in education and clinical training remain evidence-based, ethical, and interoperable within broader health care data ecosystems.

Interpretation of these findings should be approached cautiously, as the analyses focus on patterns of answer correctness rather than an evaluation of underlying clinical reasoning. Accordingly, conclusions regarding interpretability are limited to observed performance outcomes within this dataset.

### Limitations

When interpreting our findings, it is important to note that comparisons with prior LLM-USMLE benchmarking studies are inherently constrained by methodological heterogeneity, including differences in datasets, question formats, model versions, prompting strategies, and evaluation metrics. Accordingly, references to alignment or contrast with previous studies throughout the Discussion section are intended to reflect general performance trends rather than direct effect size comparisons or strict methodological equivalence.

One key limitation of this study is that the evaluation was conducted using the free, publicly available versions of each AI model, rather than their advanced or premium versions, which may offer enhanced features, faster processing, or improved accuracy. As such, the results may not fully reflect the optimal performance capabilities of these AI systems, particularly in complex medical reasoning and clinical decision-making scenarios.

Second, the assessment was based on a single set of 119 standardized USMLE-style questions, with varying numbers of questions per subject. This limited sample may not adequately represent the full breadth and depth of clinical knowledge required in real-world practice. Furthermore, subjects with fewer questions may have skewed results, limiting generalizability across disciplines.

Third, this study did not include external validation by domain experts to evaluate the accuracy or clinical relevance of AI-generated explanations. Accuracy was assessed solely based on the selected multiple-choice answer, without analyzing the underlying reasoning or clinical logic used by the models. As such, the findings reflect correctness rather than depth of understanding. Incorporating expert review in future work could help assess the quality of AI reasoning, identify reasoning discrepancies across models, and better evaluate their potential as clinical decision-support or educational tools.

Fourth, the USMLE Step 1 free 120-question sample set (January 2024 version) provided by the NBME official website is a single practice form. It may not represent a full-scale USMLE Step 1 exam distribution and randomization of different questions. In addition, the exclusive focus on a US-based licensing exam may limit the generalizability of these findings to other international or specialty examinations, such as the Professional and Linguistic Assessments Board Test, Australian Medical Council, or Membership of the Royal Colleges of Physicians. Future research should include multiple exam formats and regional assessments to better capture the global applicability and cross-context performance of AI models in medical education. A further limitation is the potential for data contamination, as some LLMs may have been trained on publicly available examination questions, meaning that observed performance may partially reflect prior exposure rather than de novo reasoning; this represents a generic challenge when benchmarking proprietary LLMs on public examination materials.

Moreover, AI performance was measured in a static, noninteractive format using a fixed prompt cycle. This design does not capture how these tools might perform in more dynamic, real-time learning environments such as tutoring platforms or patient simulations. Additionally, the use of a standardized and minimalistic prompt (“Are you sure?” repeated twice) may have constrained the models’ ability to demonstrate deeper reasoning or adaptive dialogue. Such a prompt may also differentially affect models depending on their architectural design or safety policies, potentially favoring systems optimized for self-reflection and iterative reasoning while disadvantaging models engineered for response stability or constrained by safeguards limiting self-contradiction. This approach was intentionally chosen to maintain realism and standardization across models, simulating how typical students without prompt-engineering expertise might interact with AI tools. Future studies should explore more flexible, role-based, or context-rich prompts to better evaluate reasoning depth and ecological validity.

Finally, given the rapid pace of AI development, the capabilities of these models are constantly evolving. Results obtained during this snapshot in time may not reflect future performance, underscoring the importance of ongoing evaluation and adaptation of AI tools in medical education.

### Conclusion

This study demonstrates the varying capabilities of modern AI models in addressing standardized medical exam content. In our dataset, Grok stands out for its accuracy and consistency, particularly in handling image- and case-based questions. While ChatGPT remains a dependable resource, emerging models like Grok and Copilot show increasing promise. DeepSeek, despite its visual limitations, shows strong potential in text-based domains. As AI tools continue to evolve, regular benchmarking will be essential to inform their optimal use in medical education. This comparative analysis of AI tools contributes to the field of medical internet research and informatics by providing insights into how emerging language models perform in medical knowledge assessment, supporting the development of reliable, evidence-based applications of AI in health care education and digital learning environments.

## Appendix

Multimedia Appendix 1 A representative example of the prompt used in the study, along with the full responses generated by each of the 5 evaluated AI models: ChatGPT-4, Copilot, DeepSeek, Gemini, and Grok. The responses are presented as they were outputted during the first attempt and reflect the models’ reasoning and formatting. This example illustrates differences in response accuracy, structure, and interpretation, offering insight into how each model handles standardized medical questions.

## Abbreviations

AI: artificial intelligence
LLM: large language model
NBME: National Board of Medical Examiners
USMLE: United States Medical Licensing Examination
