# Supplementary material for: Performance of 5 AI Models on United States Medical Licensing Examination Step 1 Questions: Comparative Observational Study
Source: JMIR AI. 2026 Mar 9;5:e76928. doi: 10.2196/76928 (PMC13010076; doi:10.2196/76928)
Supplement: Multimedia Appendix 1 [file ai_v5i1e76928_app1.doc]

**Supplementary Material 1. Sample Prompt and Comparative AI Model Responses**

This supplementary material provides a representative example of the prompt used in the study, along with the full responses generated by each of the five evaluated AI models: ChatGPT-4, Copilot, DeepSeek, Gemini, and Grok. The responses are presented as they were outputted during the first attempt and reflect the models’ reasoning and formatting. This example illustrates differences in response accuracy, structure, and interpretation, offering insight into how each model handles standardized medical questions.

**The question used in this example:**

A 26-year-old man comes to the office because of a 1-week history of increased urinary frequency accompanied by excessive thirst. He says he has been urinating hourly. Physical examination shows no abnormalities. Serum chemistry studies are within the reference ranges. Urine osmolality is 50 mOsmol/kg H2O. After administration of ADH (vasopressin), his urine osmolality is within the reference range. The most likely cause of this patient's symptoms is dysfunction of which of the following structures?

(A) Anterior pituitary gland

(B) Bowman capsule

(C) Glomerulus

(D) Hypophysial portal system

(E) Loop of Henle

(F) Supraoptic nucleus

**Correct Answer:** (F) Supraoptic nucleus

**ChatGPT**

| This is a board level exam for physicians. Read the question carefully and choose the correct answer.  A 26-year-old man comes to the office because of a 1-week history of increased urinary frequency accompanied by excessive thirst. He says he has been urinating hourly. Physical examination shows no abnormalities. Serum chemistry studies are within the reference ranges. Urine osmolality is 50 mOsmol/kg H2O. After administration of ADH (vasopressin), his urine osmolality is within the reference range. The most likely cause of this patient's symptoms is dysfunction of which of the following structures?  (A) Anterior pituitary gland  (B) Bowman capsule  (C) Glomerulus  (D) Hypophysial portal system  (E) Loop of Henle  (F) Supraoptic nucleus | |  |
| --- | --- | --- |
| Prompt | |  |
|  | The correct answer is:  **(F) Supraoptic nucleus**  **Explanation:**  This patient presents with **polyuria (frequent urination), polydipsia (excessive thirst), and very low urine osmolality (50 mOsm/kg H₂O)**, which indicates **diabetes insipidus (DI)**.  There are two types of diabetes insipidus:   1. **Central DI** – caused by impaired production or secretion of **antidiuretic hormone (ADH, vasopressin)**. 2. **Nephrogenic DI** – caused by renal resistance to ADH.   The key finding in this question is that **after administration of ADH, the patient’s urine osmolality normalizes**, meaning the kidneys respond to ADH. This confirms **central diabetes insipidus**, which is due to a problem with ADH production.  **Why the Supraoptic Nucleus?**  ADH is primarily **produced by the supraoptic nucleus** of the hypothalamus (with some contribution from the paraventricular nucleus) and then stored and released by the posterior pituitary. Damage to the supraoptic nucleus (e.g., trauma, tumors, inflammation) can lead to **ADH deficiency**, causing central DI.  **Why Not the Other Options?**   - **(A) Anterior pituitary gland** → Incorrect. The anterior pituitary does not produce ADH (it releases hormones like ACTH, TSH, GH, etc.). - **(B) Bowman capsule** → Incorrect. This is part of the glomerulus in the kidney and does not regulate ADH. - **(C) Glomerulus** → Incorrect. The glomerulus filters blood but does not affect ADH production. - **(D) Hypophysial portal system** → Incorrect. This transports hormones between the hypothalamus and anterior pituitary, but does not affect ADH release. - **(E) Loop of Henle** → Incorrect. While the loop of Henle plays a role in urine concentration, it does not produce ADH.   **Conclusion**  The **supraoptic nucleus** is responsible for ADH production, and its dysfunction leads to **central diabetes insipidus**, which explains this patient’s symptoms and response to vasopressin. | |
|  | ChatGPT | |
| Are you sure? | |  |
| Prompt | |  |
|  | Yes, I am sure. Let's go over the key points again to confirm.  **Breakdown of the Case:**   - **Polyuria & Polydipsia** → Suggests a disorder affecting water balance. - **Very Low Urine Osmolality (50 mOsm/kg H₂O)** → Indicates **inability to concentrate urine**. - **Correction with ADH (vasopressin)** → Means the kidneys can respond to ADH, confirming **Central Diabetes Insipidus (DI)** (not nephrogenic DI).   **Where is ADH Produced?**  ADH (vasopressin) is:   - **Synthesized** in the **supraoptic nucleus** (mostly) and paraventricular nucleus of the hypothalamus. - **Stored and released** from the **posterior pituitary**.   Since ADH is **not being produced adequately** in this case, the problem originates in the **supraoptic nucleus**, making **(F) Supraoptic nucleus** the correct answer.  Would you like a deeper dive into any specific part of the explanation? 😊 | |
|  | ChatGPT | |
| Are you sure? | |  |
| Prompt | |  |
|  | Yes, I am absolutely sure! Let's break it down once more to eliminate any doubt.  **Key Points in the Question**   1. **Polyuria (frequent urination) & Polydipsia (excessive thirst)** → Suggests an issue with water regulation. 2. **Very low urine osmolality (50 mOsm/kg H₂O)** → The kidneys are **not concentrating urine**, which is highly suggestive of **diabetes insipidus (DI)**. 3. **Response to ADH (vasopressin) → Normalization of urine osmolality**    - If the kidneys were resistant to ADH (as in nephrogenic DI), giving ADH wouldn’t help.    - Since urine osmolality **increases** after ADH, this confirms **Central DI**, meaning the body **wasn’t producing enough ADH** in the first place.   **What Produces ADH?**   - ADH (vasopressin) is **synthesized** in the **supraoptic nucleus** of the hypothalamus (with some contribution from the paraventricular nucleus). - It is then **stored and secreted** by the **posterior pituitary**. - If the **supraoptic nucleus is damaged**, ADH is not produced, leading to **Central Diabetes Insipidus**.   **Why the Other Answers Are Wrong?**   - **(A) Anterior pituitary gland** → Incorrect. The anterior pituitary **does NOT produce ADH**; it releases hormones like ACTH, TSH, GH, etc. - **(B) Bowman capsule** → Incorrect. This is part of the nephron involved in filtration, not ADH regulation. - **(C) Glomerulus** → Incorrect. The glomerulus filters plasma but does not regulate water balance directly. - **(D) Hypophysial portal system** → Incorrect. This transports hormones between the hypothalamus and anterior pituitary but does not affect ADH. - **(E) Loop of Henle** → Incorrect. The loop of Henle helps with urine concentration but does not produce ADH.   **Final Answer: (F) Supraoptic nucleus**  Since this nucleus is responsible for producing ADH, its dysfunction results in **Central Diabetes Insipidus**, explaining the patient's symptoms and response to ADH administration.  I am 100% confident in this answer! ✅ | |
|  | ChatGPT | |

**Copilot**

| This is a board level exam for physicians. Read the question carefully and choose the correct answer.  A 26-year-old man comes to the office because of a 1-week history of increased urinary frequency accompanied by excessive thirst. He says he has been urinating hourly. Physical examination shows no abnormalities. Serum chemistry studies are within the reference ranges. Urine osmolality is 50 mOsmol/kg H2O. After administration of ADH (vasopressin), his urine osmolality is within the reference range. The most likely cause of this patient's symptoms is dysfunction of which of the following structures?  (A) Anterior pituitary gland  (B) Bowman capsule  (C) Glomerulus  (D) Hypophysial portal system  (E) Loop of Henle  (F) Supraoptic nucleus | |  |
| --- | --- | --- |
| Prompt | |  |
|  | The correct answer is (F) Supraoptic nucleus.  Here's the reasoning: the patient's symptoms of excessive thirst (polydipsia) and frequent urination (polyuria) accompanied by a very low urine osmolality point to diabetes insipidus, specifically central diabetes insipidus. This condition occurs due to insufficient production or secretion of antidiuretic hormone (ADH), also known as vasopressin, which is produced in the hypothalamus—primarily by the supraoptic and paraventricular nuclei—and stored in the posterior pituitary gland.  After the administration of ADH, the patient's urine osmolality normalizes, confirming that his kidneys are functioning properly and the issue lies in the production of ADH. Dysfunction of the supraoptic nucleus, where ADH is synthesized, is the most likely cause of his symptoms.  If you have more questions about medical topics or need further clarification, let me know! | |
|  | Copilot | |
| Are you sure? | |  |
| Prompt | |  |
|  | Yes, I’m confident in my response! The clinical scenario strongly suggests central diabetes insipidus due to low urine osmolality that normalizes after ADH administration. This indicates a problem in ADH production or secretion, which involves the **supraoptic nucleus** in the hypothalamus.  However, I’m here to dig deeper if you’d like. If you feel there’s another angle to explore or something unclear, let me know—we can work through it together! | |
|  | Copilot | |
| Are you sure? | |  |
| Prompt | |  |
|  | Yes, I’m confident in my answer! The presentation of symptoms—low urine osmolality that improves after ADH administration—is characteristic of central diabetes insipidus. This points to impaired production of ADH in the hypothalamus, specifically the **supraoptic nucleus** where ADH is synthesized. The other options don’t align as well with the described findings.  However, I’m happy to revisit the question if you’d like to explore alternative perspectives or discuss the reasoning further. What’s on your mind? | |
|  | Copilot | |

**DeepSeek**

| This is a board level exam for physicians. Read the question carefully and choose the correct answer.  A 26-year-old man comes to the office because of a 1-week history of increased urinary frequency accompanied by excessive thirst. He says he has been urinating hourly. Physical examination shows no abnormalities. Serum chemistry studies are within the reference ranges. Urine osmolality is 50 mOsmol/kg H2O. After administration of ADH (vasopressin), his urine osmolality is within the reference range. The most likely cause of this patient's symptoms is dysfunction of which of the following structures?  (A) Anterior pituitary gland  (B) Bowman capsule  (C) Glomerulus  (D) Hypophysial portal system  (E) Loop of Henle  (F) Supraoptic nucleus | |  |
| --- | --- | --- |
| Prompt | |  |
|  | Let's analyze the question step-by-step to arrive at the correct answer.  **Key Clinical Findings:**   1. **Symptoms:** Polyuria (increased urinary frequency) and polydipsia (excessive thirst). 2. **Urine osmolality:** Very low (50 mOsmol/kg H2O), indicating dilute urine. 3. **Response to ADH (vasopressin):** Urine osmolality normalizes after administration, suggesting the kidneys can respond to ADH. 4. **Serum chemistry:** Normal (rules out hyperglycemia, hypercalcemia, or other metabolic causes of polyuria).   **Diagnosis:**   - The patient has **central diabetes insipidus (DI)**, a condition where the **hypothalamus or posterior pituitary fails to produce or secrete adequate ADH (vasopressin)**. - The **low urine osmolality** suggests inability to concentrate urine due to lack of ADH. - The **normalization of urine osmolality after ADH administration** confirms that the renal tubules (nephron) are responsive to ADH, localizing the defect to **ADH production/secretion**, not the kidneys (nephrogenic DI).   **Structure Responsible:**   - ADH is synthesized in the **supraoptic nucleus (and paraventricular nucleus)** of the **hypothalamus** and transported to the **posterior pituitary** for release. - Dysfunction of the **supraoptic nucleus** (choice F) would impair ADH production, leading to central DI.   **Why Not the Other Options?**   - **(A) Anterior pituitary gland:** Does not produce ADH (ADH is secreted by the posterior pituitary). - **(B) Bowman capsule / (C) Glomerulus:** Dysfunction would cause proteinuria or hematuria, not isolated polyuria with dilute urine. - **(D) Hypophysial portal system:** Carries hypothalamic hormones to the anterior pituitary, not ADH (which travels via axonal transport to the posterior pituitary). - **(E) Loop of Henle:** Dysfunction would impair urine concentration, but the patient responds to ADH, ruling out a primary renal defect.   **Conclusion:**  The most likely cause is dysfunction of the **supraoptic nucleus**, which produces ADH.  **Correct Answer: (F) Supraoptic nucleus** | |
|  | DeepSeek | |
| Are you sure? | |  |
| Prompt | |  |
|  | Yes, I am confident that the correct answer is **(F) Supraoptic nucleus**. Let me explain why this is the best choice and why the other options are incorrect.  **Reaffirming the Diagnosis: Central Diabetes Insipidus (DI)**  The patient has:   - **Polyuria (excessive urination) and polydipsia (excessive thirst)**—classic symptoms of diabetes insipidus (DI). - **Dilute urine (osmolality = 50 mOsm/kg H₂O)**—indicating inability to concentrate urine. - **Normal response to exogenous ADH (urine osmolality normalizes)**—proving the kidneys can respond to ADH, ruling out nephrogenic DI. - **Normal serum chemistry**—excluding osmotic diuresis (e.g., hyperglycemia).   This confirms **central DI**, where the problem is **insufficient ADH production or secretion**, not kidney resistance to ADH.  **Why the Supraoptic Nucleus (F)?**   - ADH (vasopressin) is synthesized in **neurons of the supraoptic and paraventricular nuclei of the hypothalamus**. - These neurons send axons to the **posterior pituitary**, where ADH is stored and released. - Dysfunction in the **supraoptic nucleus** (e.g., due to trauma, infiltrative disease, or idiopathic causes) would impair ADH production, leading to central DI.   **Why Not the Other Options?**  Let’s revisit why the other structures are **not** the primary issue:   | **Option** | **Why Incorrect?** | | --- | --- | | **(A) Anterior pituitary gland** | ADH is produced in the hypothalamus and released from the **posterior pituitary**, not the anterior pituitary. Anterior pituitary dysfunction would cause issues with ACTH, TSH, GH, etc., but not ADH deficiency. | | **(B) Bowman capsule** | Part of the glomerular filtration apparatus; dysfunction would cause hematuria, proteinuria, or renal failure, not isolated polyuria with dilute urine. | | **(C) Glomerulus** | Dysfunction would lead to filtration problems (e.g., nephritic/nephrotic syndrome), not pure water imbalance. | | **(D) Hypophysial portal system** | Carries hypothalamic releasing hormones (e.g., CRH, TRH) to the **anterior pituitary**, but ADH is transported via neuronal axons to the posterior pituitary. This system is irrelevant to ADH regulation. | | **(E) Loop of Henle** | Critical for urine concentration, but since the patient **responds to ADH**, the defect is not in the kidney. Dysfunction here would cause **nephrogenic DI**, not central DI. |   **Final Confirmation**  The patient’s **response to ADH** confirms the problem is **ADH deficiency**, not kidney dysfunction. The **supraoptic nucleus** is the most likely site of pathology.  **Correct Answer: (F) Supraoptic nucleus ✅**  Would you like any further clarification on a specific part? | |
|  | DeepSeek | |
| Are you sure? | |  |
| Prompt | |  |
|  | Yes, I am absolutely sure the correct answer is **(F) Supraoptic nucleus**. Let me address any remaining doubts by reinforcing the logic and ruling out alternatives even more rigorously.  **Why This is Central Diabetes Insipidus (DI)**   1. **Key Findings:**    - Polyuria + polydipsia + **very dilute urine (50 mOsm/kg)** → Suggests impaired urine concentration.    - **Normalizes with ADH** → Kidneys can respond to ADH, so the defect is **not in the kidney** (rules out nephrogenic DI).    - Normal serum electrolytes → No osmotic diuresis (e.g., diabetes mellitus).   **Conclusion:** The problem is **ADH deficiency** (central DI), not kidney resistance or osmotic diuresis.  **Why the Supraoptic Nucleus?**   - **ADH is produced in the hypothalamus** (specifically the **supraoptic and paraventricular nuclei**). - These neurons send axons to the **posterior pituitary**, where ADH is released into the bloodstream. - **Dysfunction here (e.g., trauma, tumors, idiopathic)** → No ADH production → Central DI.   **This is the *only* structure in the options responsible for ADH synthesis.**  **Why the Other Options Are *Definitively* Wrong**   | **Option** | **Flaw in Reasoning** | | --- | --- | | **(A) Anterior pituitary** | Doesn’t make ADH. Anterior pituitary makes ACTH, TSH, etc. ADH comes from the *posterior* pituitary (but is made in the hypothalamus). | | **(B) Bowman capsule/ (C) Glomerulus** | These are part of filtration. Dysfunction would cause proteinuria, hematuria, or renal failure—*not* isolated dilute polyuria. | | **(D) Hypophysial portal system** | Carries hypothalamic hormones (e.g., CRH, GnRH) to the *anterior* pituitary. ADH transport doesn’t use this system (it uses axonal transport). | | **(E) Loop of Henle** | Critical for countercurrent multiplication, but since the patient **responds to ADH**, the kidney is fine. Loop dysfunction would cause *nephrogenic* DI (no response to ADH). |   **Common Pitfall: Confusing Posterior vs. Anterior Pituitary**   - Some might think the **anterior pituitary (A)** is involved because it’s part of the "pituitary gland," but ADH is *only* produced in the hypothalamus and released from the *posterior* pituitary. - The **supraoptic nucleus (F)** is the *only* option that directly explains ADH deficiency.   **Final Answer: (F) Supraoptic nucleus**  This is the **only anatomically and physiologically correct answer** based on the patient’s lab results and response to ADH.  If you’re still hesitant, consider:   - **What else could cause central DI?** Only hypothalamic (supraoptic nucleus) or posterior pituitary damage. The latter isn’t an option here. - **Why not the kidney?** The normal response to ADH rules it out.   Would you like a reference from a textbook (e.g., *Guyton and Hall Physiology* or *Harrison’s*) to confirm? | |
|  | DeepSeek | |

**Gemini**

| This is a board level exam for physicians. Read the question carefully and choose the correct answer.  A 26-year-old man comes to the office because of a 1-week history of increased urinary frequency accompanied by excessive thirst. He says he has been urinating hourly. Physical examination shows no abnormalities. Serum chemistry studies are within the reference ranges. Urine osmolality is 50 mOsmol/kg H2O. After administration of ADH (vasopressin), his urine osmolality is within the reference range. The most likely cause of this patient's symptoms is dysfunction of which of the following structures?  (A) Anterior pituitary gland  (B) Bowman capsule  (C) Glomerulus  (D) Hypophysial portal system  (E) Loop of Henle  (F) Supraoptic nucleus | |  |
| --- | --- | --- |
| Prompt | |  |
|  | Let's analyze the case and each option:   - **Patient Presentation:**   - Increased urinary frequency (polyuria)   - Excessive thirst (polydipsia)   - Low urine osmolality (dilute urine)   - Normal serum chemistry   - Response to ADH (vasopressin) with increased urine osmolality - **Interpretation:**   - The patient's symptoms suggest diabetes insipidus, a condition characterized by the inability to concentrate urine.   - The fact that his urine osmolality increased after ADH administration indicates that his kidneys are capable of responding to ADH, meaning the problem is a deficiency of ADH itself.   - ADH is produced in the Supraoptic and Paraventricular nuclei of the Hypothalamus, and transported to the posterior pituitary. ^1^   [1. app.usmlepreps.com](https://app.usmlepreps.com/ViewQuestion/question/214-A-29-year-old-man-comes-to-the-office-due-to-progressive-headaches-fatigue-and-visual-disturbances-for-the-past-6-weeks-nbsp-He-was-diagnosed-with-sarcoidosis-a-year-ago-after-having-a-dry-cough-and-hypercalcemia-nbsp-The-patient-39-s-condition-was-treated-with-oral-glucocorticoid-therapy-which-was-discontinued-after-6-months-due-to-improvement-in-symptoms-nbsp-Physical-examination-reveals-bitemporal-defects-on-visual-field-testing-but-is-otherwise-normal-nbsp-Laboratory-studies-again-show-mild-hypercalcemia-but-the-remainder-of-the-values-including-complete-blood-count-serum-glucose-creatinine-and-electrolytes-are-within-normal-reference-ranges-nbsp-MRI-of-the-brain-reveals-a-soft-tissue-mass-involving-the-hypothalamus-and-pituitary-stalk-nbsp-Which-of-the-following-pituitary-hormones-is-most-likely-elevated-in-this-patient-) - **Analysis of Options:**   - **(A) Anterior pituitary gland:** The anterior pituitary primarily releases hormones like ACTH, TSH, GH, and prolactin. While it plays a role in overall hormonal balance, it's not directly responsible for ADH production.   - **(B) Bowman capsule:** The Bowman capsule is part of the renal corpuscle, involved in filtering blood. While glomerular filtration is essential for urine formation, it's not the primary site of ADH action.   - **(C) Glomerulus:** Similar to the Bowman capsule, the glomerulus is involved in blood filtration, not ADH production or release.   - **(D) Hypophysial portal system:** This system connects the hypothalamus to the anterior pituitary, not the posterior pituitary where ADH is released.   - **(E) Loop of Henle:** The loop of Henle is crucial for establishing the concentration gradient in the kidneys, allowing for urine concentration. However, it requires ADH to function properly. The problem in this case is the lack of ADH, not a dysfunction of the loop of henle.   - **(F) Supraoptic nucleus:** This is the location in the hypothalamus where ADH is synthesized. Dysfunction here would lead to a deficiency in ADH production, resulting in the observed symptoms. - **Conclusion:**   - The most likely cause is a dysfunction in the supraoptic nucleus, which is responsible for ADH production.   Therefore, the correct answer is (F). | |
|  | Gemini | |
| Are you sure? | |  |
| Prompt | |  |
|  | Yes, I am sure. Let's reiterate the reasoning:   - **Central Diabetes Insipidus:** The patient's response to ADH (vasopressin) confirms central diabetes insipidus. This means the problem lies in the production or release of ADH, not in the kidney's response to it. - **ADH Production:** ADH is synthesized in the supraoptic and paraventricular nuclei of the hypothalamus. - **Posterior Pituitary Release:** ADH is then transported to the posterior pituitary for release. - **Dysfunction Location:** Therefore, the dysfunction must be in the hypothalamus (specifically the supraoptic nucleus or paraventricular nucleus, with the supraoptic being listed as an option), or in the transport system between the hypothalamus and the posterior pituitary. - **Exam Choices:** Of the given options, only the supraoptic nucleus directly relates to ADH production.   Therefore, the only correct answer from the provided options is (F) Supraoptic nucleus. | |
|  | Gemini | |
| Are you sure? | |  |
| Prompt | |  |
|  | Yes, I am confident in my answer. The patient's clinical presentation, including polyuria, polydipsia, low urine osmolality, and a positive response to ADH administration, strongly suggests central diabetes insipidus. This condition is caused by a deficiency in ADH production or release.  Here's a breakdown of why the supraoptic nucleus is the correct answer:   - **ADH Production:** The supraoptic and paraventricular nuclei of the hypothalamus are the sites of ADH synthesis. - **Central Diabetes Insipidus:** The fact that the patient responded to exogenous ADH indicates that the kidneys are functioning correctly. The problem lies in the production or release of ADH. - **Location of Dysfunction:** Therefore, the dysfunction must be located in the hypothalamus, where ADH is produced, or in the pathway that transports ADH to the posterior pituitary. - **Exam Options:** Among the provided options, only the supraoptic nucleus directly relates to ADH production.   Therefore, the most likely cause of the patient's symptoms is dysfunction of the supraoptic nucleus. | |
|  | Gemini | |

**Grok**

| This is a board level exam for physicians. Read the question carefully and choose the correct answer.  A 26-year-old man comes to the office because of a 1-week history of increased urinary frequency accompanied by excessive thirst. He says he has been urinating hourly. Physical examination shows no abnormalities. Serum chemistry studies are within the reference ranges. Urine osmolality is 50 mOsmol/kg H2O. After administration of ADH (vasopressin), his urine osmolality is within the reference range. The most likely cause of this patient's symptoms is dysfunction of which of the following structures?  (A) Anterior pituitary gland  (B) Bowman capsule  (C) Glomerulus  (D) Hypophysial portal system  (E) Loop of Henle  (F) Supraoptic nucleus | |  |
| --- | --- | --- |
| Prompt | |  |
|  | Let’s break this down step-by-step to determine the correct answer.  The patient is a 26-year-old man presenting with a 1-week history of increased urinary frequency and excessive thirst, urinating hourly. These symptoms suggest polyuria (excessive urine output) and polydipsia (excessive thirst), which are classic signs of a condition affecting water balance, such as diabetes insipidus or diabetes mellitus. Physical examination shows no abnormalities, and serum chemistry studies (e.g., glucose, electrolytes) are normal, ruling out diabetes mellitus (which would show elevated blood glucose). The key diagnostic clues are the urine osmolality of 50 mOsmol/kg H2O (very low, indicating dilute urine) and the response to ADH (vasopressin), after which urine osmolality normalizes.  Urine osmolality of 50 mOsmol/kg H2O is significantly below the normal range (typically 300–900 mOsmol/kg H2O when concentrated), indicating the kidneys are not concentrating urine despite the patient’s thirst, which suggests dehydration. This points to a problem with water reabsorption. The fact that urine osmolality normalizes after ADH administration tells us the kidneys can respond to ADH by concentrating urine, meaning the issue is not in the kidneys’ ability to respond (nephrogenic diabetes insipidus is ruled out). Instead, the problem lies in the body’s production or release of ADH, pointing to central diabetes insipidus.  Central diabetes insipidus occurs when the posterior pituitary fails to release sufficient ADH, often due to a dysfunction in the hypothalamus, where ADH is synthesized. The supraoptic nucleus (and paraventricular nucleus) in the hypothalamus produces ADH, which is then transported to and stored in the posterior pituitary for release. If the supraoptic nucleus is dysfunctional, ADH production is impaired, leading to dilute urine and symptoms like those described. The normalization of urine osmolality with exogenous ADH confirms the kidneys are functional and the issue is upstream, in ADH production.  Now, let’s evaluate the options:   - **(A) Anterior pituitary gland**: The anterior pituitary produces hormones like ACTH, TSH, and FSH, but not ADH. ADH is made in the hypothalamus and released by the posterior pituitary. This is incorrect. - **(B) Bowman capsule**: This is part of the nephron where filtration occurs, not directly involved in urine concentration or ADH response. Incorrect. - **(C) Glomerulus**: This filters blood into the nephron but doesn’t regulate water reabsorption or respond to ADH. Incorrect. - **(D) Hypophysial portal system**: This connects the hypothalamus to the anterior pituitary, regulating anterior pituitary hormones, not ADH release from the posterior pituitary. Incorrect. - **(E) Loop of Henle**: This contributes to urine concentration but isn’t directly responsible for ADH production or response. The kidneys work here, as shown by the ADH response. Incorrect. - **(F) Supraoptic nucleus**: This hypothalamic nucleus synthesizes ADH. Dysfunction here would reduce ADH production, causing dilute urine (low osmolality) and polyuria, with normalization after exogenous ADH. This fits perfectly.   The most likely cause is dysfunction of the supraoptic nucleus, leading to central diabetes insipidus.  **Correct answer: (F) Supraoptic nucleus** | |
|  | Grok | |
| Are you sure? | |  |
| Prompt | |  |
|  | Let’s double-check the reasoning to ensure confidence in the answer.  The patient has polyuria (frequent urination, hourly for a week) and polydipsia (excessive thirst), which are hallmarks of a water balance disorder. The physical exam is normal, and serum chemistry studies are within reference ranges, so there’s no evidence of hyperglycemia (ruling out diabetes mellitus) or electrolyte imbalances. The critical lab findings are: urine osmolality is 50 mOsmol/kg H2O (very low, indicating dilute urine), and after ADH (vasopressin) administration, urine osmolality rises to the reference range (normalizes).  Low urine osmolality despite thirst (implying dehydration) suggests the kidneys aren’t concentrating urine properly. Normally, dehydration triggers ADH release to increase water reabsorption in the kidneys’ collecting ducts, concentrating the urine. Here, the baseline urine is dilute, so either ADH isn’t present (central diabetes insipidus) or the kidneys can’t respond to it (nephrogenic diabetes insipidus). The key is the ADH test: when exogenous ADH is given, urine osmolality normalizes, meaning the kidneys *can* respond to ADH. This rules out nephrogenic diabetes insipidus (where the kidneys are defective) and points to a lack of endogenous ADH production or release—central diabetes insipidus.  In central diabetes insipidus, the issue is insufficient ADH from the hypothalamic-pituitary axis. ADH is synthesized in the supraoptic and paraventricular nuclei of the hypothalamus, transported along axons, and stored/released by the posterior pituitary. The question asks for the dysfunctional structure. Let’s revisit the options with this in mind:   - **(A) Anterior pituitary gland**: This produces hormones like growth hormone and prolactin, not ADH. ADH comes from the posterior pituitary, but its synthesis is hypothalamic. This doesn’t fit. - **(B) Bowman capsule**: Part of the nephron’s filtration unit, unrelated to ADH production or action on urine concentration. No. - **(C) Glomerulus**: Filters blood into the nephron, not involved in ADH-mediated water reabsorption. No. - **(D) Hypophysial portal system**: Links the hypothalamus to the anterior pituitary for regulating its hormones, not involved in ADH (posterior pituitary pathway). No. - **(E) Loop of Henle**: Helps establish the medullary concentration gradient, but water reabsorption occurs in the collecting ducts under ADH control. The kidneys respond to ADH here, so this isn’t the issue. - **(F) Supraoptic nucleus**: A hypothalamic nucleus that produces ADH. If dysfunctional, ADH synthesis drops, leading to dilute urine (low osmolality) and polyuria. Exogenous ADH bypasses this defect, normalizing osmolality, which matches the scenario.   Could it be the posterior pituitary instead? The posterior pituitary stores and releases ADH, but the question lists “supraoptic nucleus” as an option, not “posterior pituitary.” Dysfunction in the supraoptic nucleus (where ADH is made) would prevent ADH from reaching the posterior pituitary, fitting the symptoms and test results. In medical contexts, central diabetes insipidus is often tied to hypothalamic dysfunction (e.g., trauma, tumors), and the supraoptic nucleus is a precise answer.  The normalization after ADH administration is the clincher: it confirms the kidneys are fine, and the defect is in ADH production. Among the options, only the supraoptic nucleus aligns with this pathophysiology.  Yes, I’m sure. **Correct answer: (F) Supraoptic nucleus**  *Disclaimer: Grok is not a doctor; please consult one. Don't share information that can identify you.* | |
|  | Grok | |
| Are you sure? | |  |
| Prompt | |  |
|  | I understand your hesitation—let’s triple-check to be absolutely certain, diving deep into the details and considering any possible oversight.  The patient, a 26-year-old man, has a 1-week history of urinating hourly (polyuria) and excessive thirst (polydipsia). These symptoms scream a water balance issue. Physical exam is normal, and serum chemistry studies are within reference ranges, so no diabetes mellitus (normal glucose), no electrolyte chaos, and no obvious systemic disease. The lab data: urine osmolality is 50 mOsmol/kg H2O (extremely low, normal range is ~300–900 when concentrated), meaning the urine is dilute despite thirst (a sign of dehydration). After ADH (vasopressin) is given, urine osmolality normalizes (rises to reference range). The question asks which structure’s dysfunction causes this.  Polyuria and polydipsia suggest either diabetes insipidus (central or nephrogenic) or psychogenic polydipsia. Psychogenic polydipsia (compulsive water drinking) would dilute urine, but thirst here follows polyuria (he’s compensating for water loss), and the ADH response needs explaining—so it’s unlikely. Diabetes insipidus fits better: central if ADH production/release is impaired, nephrogenic if kidneys ignore ADH.   - **Urine osmolality 50 mOsmol/kg H2O**: Normal plasma osmolality is ~285–295 mOsmol/kg H2O. If kidneys were concentrating urine in dehydration, urine osmolality should exceed plasma’s. At 50, it’s dilute—kidneys aren’t reabsorbing water. - **Post-ADH normalization**: ADH acts on collecting ducts (via V2 receptors, aquaporins) to reabsorb water. If osmolality normalizes (say, >300 mOsmol/kg H2O), the kidneys respond to ADH, ruling out nephrogenic diabetes insipidus (where kidneys are defective).   This leaves central diabetes insipidus: insufficient ADH production or release. The water deprivation test (implied here) with ADH response is classic: in central DI, urine concentrates only when ADH is provided. The defect is upstream of the kidneys, in the hypothalamic-pituitary axis.  Now, the options:   - **(A) Anterior pituitary gland**: Secretes ACTH, TSH, etc., via hypothalamic releasing hormones. ADH isn’t involved—ADH is from the posterior pituitary, synthesized in the hypothalamus. Wrong structure. - **(B) Bowman capsule**: Encases the glomerulus, filters blood. No role in ADH or urine concentration (that’s distal nephron/collecting ducts). No. - **(C) Glomerulus**: Filters plasma into the nephron. ADH acts downstream (collecting ducts), and filtration isn’t the issue here. No. - **(D) Hypophysial portal system**: Blood vessels linking hypothalamus to anterior pituitary. ADH travels via axons to the posterior pituitary, not this system. No. - **(E) Loop of Henle**: Creates the medullary gradient for concentration, but ADH directly affects collecting ducts. Kidneys respond to ADH, so the loop isn’t dysfunctional. No. - **(F) Supraoptic nucleus**: Hypothalamic nucleus (with paraventricular nucleus) where ADH is synthesized. If dysfunctional (e.g., damage, tumor), ADH production fails, causing dilute urine (low osmolality), polyuria, and thirst. Exogenous ADH fixes it by supplying what’s missing. Matches perfectly.   Could it be the posterior pituitary? It releases ADH, but the question specifies “supraoptic nucleus,” not “posterior pituitary.” If the supraoptic nucleus fails, no ADH reaches the posterior pituitary—same outcome. In board exams, precision matters, and supraoptic nucleus is the site of synthesis, often the focus in central DI etiology questions.  Alternative conditions (e.g., osmotic diuresis, renal disease) don’t fit: normal serum chemistry and ADH response rule them out. The timeline (1 week) suggests an acute hypothalamic issue (e.g., trauma, infection), but etiology isn’t asked—just the structure.  The logic holds: low ADH production (supraoptic nucleus dysfunction) → dilute urine → polyuria/polydipsia → ADH administration corrects it. No other option fits.  I’m confident: **(F) Supraoptic nucleus**.  *Disclaimer: Grok isn’t a doctor; consult one. Don’t share identifying info.* | |
|  | Grok | |
